# The secondary structural difference between Lewy body and glial cytoplasmic inclusion in autopsy brain with synchrotron FTIR micro-spectroscopy

**DOI:** 10.1038/s41598-020-76565-6

**Published:** 2020-11-10

**Authors:** Katsuya Araki, Naoto Yagi, Yuka Ikemoto, Hideki Hayakawa, Harutoshi Fujimura, Taro Moriwaki, Yoshitaka Nagai, Shigeo Murayama, Hideki Mochizuki

**Affiliations:** 1 Department of Neurology, Osaka University Graduate School of Medicine, 2-2 Yamadaoka, Suita, Osaka 565-0871 Japan; 2 Toyonaka Municipal Hospital, 4-14-1 Shibaharacho, Toyonaka, Osaka 560-8565 Japan; 3 Japan Synchrotron Radiation Research Institute (JASRI/SPring-8), 1-1-1 Kouto, Sayo, Sayo, Hyogo 679-5198 Japan; 4 Department of Neurology, Toneyama National Hospital, 5-1-1 Toneyama, Toyonaka, Osaka 560-8522 Japan; 5 Department of Neurotherapeutics, Osaka University Graduate School of Medicine, 2-2 Yamadaoka, Suita, Osaka 565-0871 Japan; 6 Department of Neuropathology, The Brain Bank for Aging Research, Tokyo Metropolitan Geriatric Hospital and Institute of Gerontology, 35-2 Sakaecho, Itabashi-ku, Tokyo, 173-0015 Japan

**Keywords:** Parkinson's disease, Neurodegenerative diseases, Structural biology, Prions, Protein aggregation

## Abstract

Lewy bodies (LBs) and glial cytoplasmic inclusions (GCIs) are specific aggregates found in Parkinson’s disease (PD) and multiple system atrophy (MSA), respectively. These aggregates mainly consist of α-synuclein (α-syn) and have been reported to propagate in the brain. In animal experiments, the fibrils of α-syn propagate similarly to prions but there is still insufficient evidence to establish this finding in humans. Here, we analysed the protein structure of these aggregates in the autopsy brains of patients by synchrotron Fourier-transform infrared micro-spectroscopy (FTIRM) analysis without extracting or artificially amplifying the aggregates. As a result, we found that the content of the β-sheet structure in LBs in patients with PD was significantly higher than that in GCIs in patients with MSA (52.6 ± 1.9% in PD vs. 38.1 ± 0.9% in MSA, *P* < 0.001). These structural differences may provide clues to the differences in phenotypes of PD and MSA.

## Introduction

Lewy bodies (LBs) are a neuropathological hallmark of Parkinson's disease (PD) and dementia with Lewy bodies (DLB)^1,2^, and glial cytoplasmic inclusions (GCIs) are the pathological hallmark of multiple system atrophy (MSA)^3^. Because both LB and GCI mainly consist of α-synuclein (α-syn)^1–3^, PD, DLB, and MSA are neurodegenerative disorders that have been pathologically classified as synucleinopathies. In animal experiments, fragments of α-syn fibrils formed in vitro and rich in β-sheets propagate in the brain and are transmitted to other individuals similarly to prions^5,6^. If α-syn similarly propagates in humans, β-sheet-rich fibrils should be detectable in the brains of patients with synucleinopathy. Owing to the small size of aggregates in the brain, structural analysis of aggregates is difficult. However, using synchrotron Fourier transform infrared micro-spectroscopy (FTIRM) which has been successfully used in the analysis of senile plaques^7–9^ and LBs^10^, we found that LBs in the brains of patients with PD indeed have a β-sheet-rich structure^10^.

Recently, by investigating the propagation of α-syn in the mouse brain using extracts from the brains of patients with PD and MSA, Prusiner et al. have suggested the possibility that the human α-syn aggregates formed in the brains of patients with PD and MSA are structurally different^4^. In the present study, to test this hypothesis, we performed synchrotron FTIRM measurements of LBs and GCIs in the autopsy brains of patients pathologically diagnosed with PD and MSA, respectively. To the best of our knowledge, this is the first study in which aggregates in the brain were directly (without extraction or artificial amplification) analysed to confirm the secondary structural differences between α-syn aggregates.

## Results

### Two dimensional FTIRM mapping of LBs and GCIs

**Figure 1:**
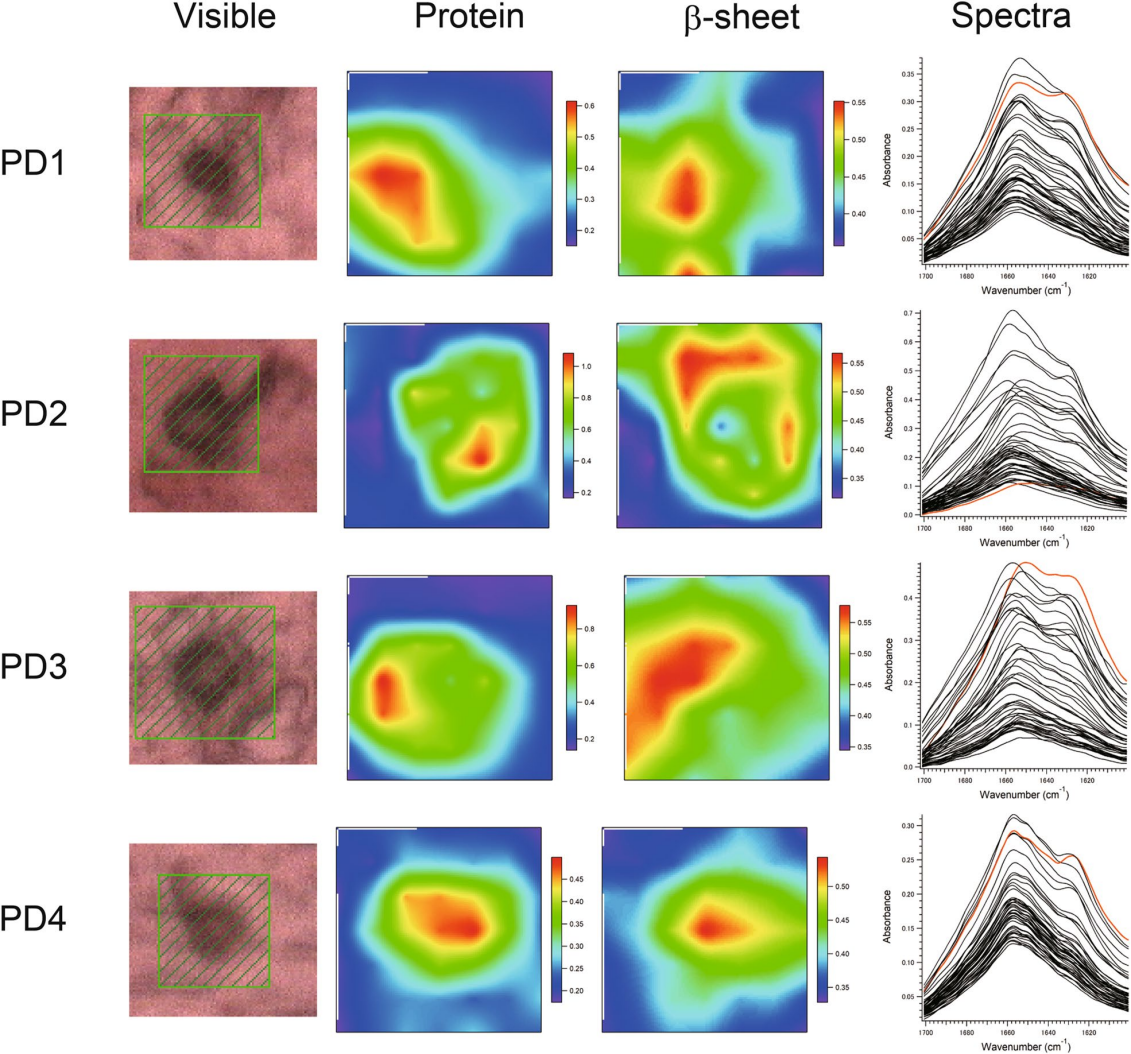
Visible and FTIR images of LBs in the medullary dorsal vagal nucleus derived from the PD patients (PD1-4, PD2-1, PD3-2, and PD4-1). Shown from left to right are the microscopy images, the amounts of total protein, the proportions of the β-sheet structure, and the spectra at all points in the scan. The colour bar indicates low (blue) to high (red) contents. The area shaded in green in the visible image was scanned with 3 µm steps. 7 × 7 points = 21 × 21 µm^2^. The red solid line in the spectra shows the FTIR spectrum of the β-sheet richest point.

**Figure 2:**
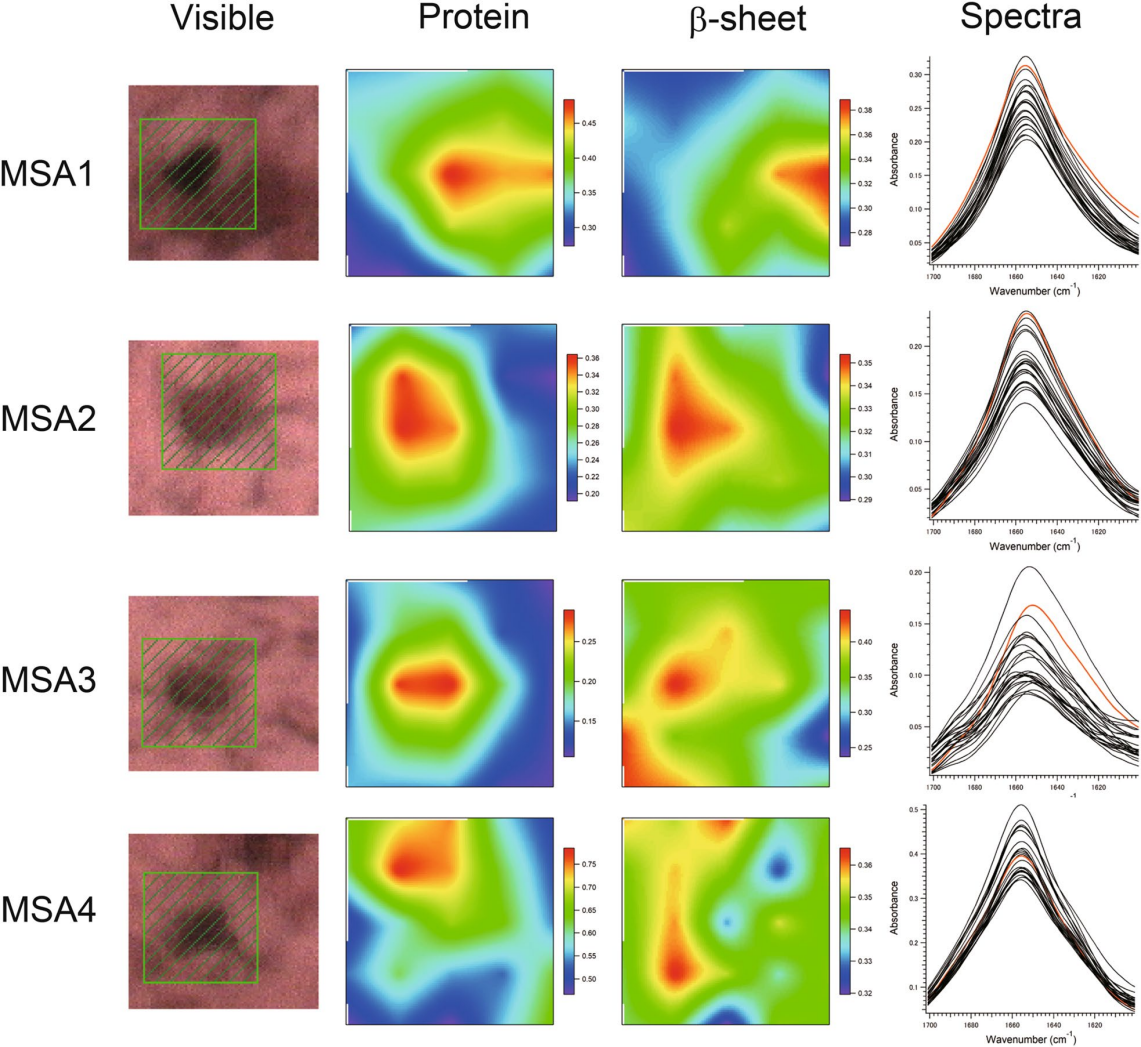
Visible and FTIR images of GCIs in the medullary dorsal vagal nucleus derived from the MSA patients (MSA1-4, MSA2-1, MSA3-4, and MSA4-1). Shown from left to right are the microscopy images, the amounts of total protein, the proportions of β-sheet structure, and the spectra at all points in the scan. The colour bar indicates low (blue) to high (red) contents. The area shaded in green in the visible image was scanned with 3 µm steps. 5 × 5 points = 15 × 15  µm^2^. The red solid line in the spectra shows the FTIR spectrum of the β-sheet richest point.

We studied brain sections that contained LBs and GCIs from four patients with PD and four with MSA (Supplementary Table S1). Four LBs or GCIs in each patient’s brain were scanned by FTIRM and the spectra obtained were analyzed as previously described^10^. Figures 1 and 2 show the two dimensional (2D) mapping of components in the FTIRM spectra for the brain sections including LBs and GCIs, respectively (see the following section for the component analysis method). The unsmoothed data are shown in Supplementary Fig. S1. The area with a large amount of protein coincides with the area that is stained by immunostaining in most cases. The result of the PD2 patient showed that the proportion of β-sheet structure in the halo is higher than that in the core, as we previously observed in some LBs^10^. On the other hand, as is the case in the MSA4 patient, the peak corresponding to a large amount of protein sometimes does not completely correspond to the area stained by immunostaining. Although this may be due to technical factors such as the uneven thickness of the section, the inclination of the section, or the shift of the beam, the exact reason is as yet unknown. Therefore, we used all the data in the statistical analysis. The spectra in Figs. 1 and 2 are those obtained at each point in the scan. It is clear that the spectra from LB and GCI are different: while the spectra of GCIs are pointed with a single peak, those of LBs have a flat top with possibly two peaks. Spectra from all other LBs and GCIs are shown in Supplementary Fig. S2 and Supplementary Fig. S3.

### Comparison of spectra between LBs and GCIs

**Figure 3:**
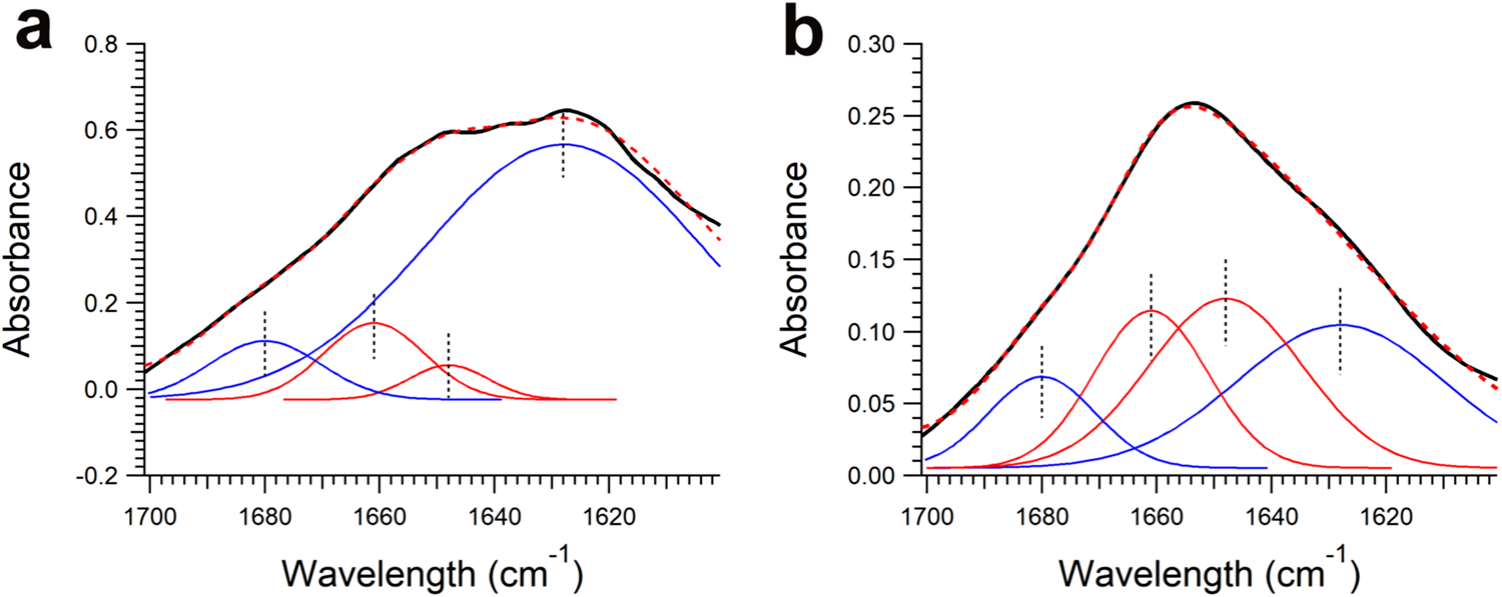
The solid black line in each panel shows a typical FTIR spectrum (amide I region) obtained from **(a)** LBs and **(b)** GCIs. Blue and red lines represent contributions of β-sheet structures and non-β-sheet structures (α-helices, random coils, and others), respectively. The dotted line represents the fitted curve. The spectra were fitted with Gaussian models centred at 1628, 1680 (β-sheets, blue line), 1648, and 1661 (α-helices, random coils, and others, red line) cm^−1^.

Figure 3 shows examples of spectra from LBs and GCIs at points where the β-sheet content was highest. The peaks were fitted with four Gaussians centered at 1628 and 1680 cm^–1^ (representing β-sheets), and 1648 and 1661 cm^–1^ (representing α-helices, random coils, and other conformations), as in our previous study^10^. The results show that LBs have a higher proportion of the β-sheet peak at 1628 cm^–1^, whereas GCIs have a higher proportion of the α-helix peak at 1648 cm^–1^.

### Comparison of proportion of β-sheet structure between LBs and GCIs

**Figure 4:**
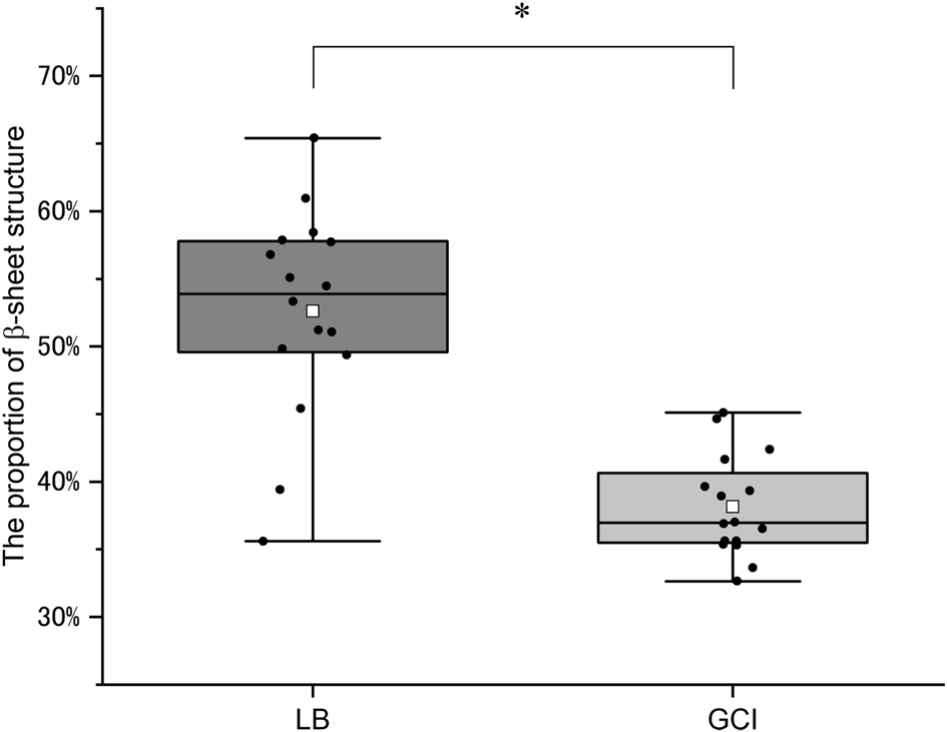
Proportion of β-sheet structures between LBs and GCIs. The white boxes represent mean values, lines represent median values, and the tops and bottoms of the boxes represent the upper and lower limits of the first and third quartiles, respectively. The ends of the whiskers represent maximum and minimum data points. Black dots are individual analysis values. The proportions of the β-sheet structure in LBs and GCIs are 52.6 ± 1.9% (n = 16) and 38.1 ± 0.9% (n = 16) (mean ± the standard error of the mean), respectively. The median values are 53.9% and 37.0%, respectively. Asterisks above the boxes indicate significant differences determined by Student’s t-test (*P < 0.001).

The proportion of the β-sheet structure was obtained by integrating areas of the two Gaussian functions representing β-sheets in the spectrum and dividing the sum by the total amide I peak area. Figure 4 shows that the proportion of the β-sheet structure in LBs was significantly higher than that in GCIs (*P* < 0.001). Considering this difference in the proportion of the β-sheet structure in these aggregates, the structures of fibrils in LBs and GCIs are considered significantly different.

## Discussion

LBs and GCIs mainly consist of α-syn and have been reported to propagate in the brain^1–4^. In animal experiments, the fibrils of α-syn propagate in a prion-like manner but there is still insufficient evidence to establish this finding in humans^5,6^. In this study, we performed synchrotron FTIRM measurements of autopsy brains with synucleinopathies and found that the proportion of the β-sheet structure in LBs was significantly higher than that in GCIs. In this study, the samples were removed within 17 h after death and quick-frozen. Then, they kept frozen in the Brain Bank until use for up to 17 years. The proportion of β-sheets in LBs is similar to that found in the previous study (43.8 to 63.8%) in which freshly obtained brain sections were chemically fixed and embedded in paraffin^10^. Thus, the difference in the sample preparation techniques does not seem to markedly affect the results. In both cases, the sections were dried just before the FTIRM measurements, which may affect the secondary structure of α-syn^11,12^. However, since all sections were prepared using the same standard procedure, there is no factor that affects LBs and GCIs differently. Thus, the difference observed in this study is considered to exist in the brains of the patients.

Many researchers have shown that LBs have a fibril-like structure, as demonstrated by EM^1–3^, but, there has never been a report that the fibril-like structure had the cross-β structure. We have recently shown by microbeam X-ray diffraction (XRD) analysis that LBs show a sharp peak derived from the cross-β structure^13^ suggesting that LBs contain amyloid fibrils. In the same study, we found that GCIs do not show a sharp peak derived from the cross-β structure. Our present finding that the proportion of the β-sheet structure in GCIs is lower than that in LBs is in agreement with the XRD result^13^. Recently, Schweighauser et al. used cryo-EM to show that GCIs included protofilaments that have cross-β hairpins^14^. Soto et al*.* found by protein misfolding cyclic amplification (PMCA) that α-syn aggregates that are associated with PD and MSA have different conformations of α-syn^15^. Taken together with our FTIR and XRD results, it seems that both LBs and GCIs have the cross β-structure but the structure of α-syn in LBs is different from that in GCIs at the stage of accumulation in the brain. The fibrils of LBs may have more β-sheet structures than those of GCIs. A large part of α-syn in GCIs may not fold into β-sheets, or GCIs may be abundant in proteins other than α-syn.

From the viewpoint of molecular science, PD seems to be a heterologous disease, and it is likely that the differences in neurological findings and rate of progression may be explained by the differences in the types of aggregates in the brain. In the future, PD may be reclassified into several diseases corresponding to the types of aggregates. The FTIRM method may help in the construction of such a disease concept.

## Methods

All experimental protocols were approved by the Ethical Review Board of the Tokyo Metropolitan Institute of Gerontology and Osaka University Graduate School of Medicine and were performed in accordance with the Ethical Guidelines for Clinical Research of the Ministry of Health, Labour and Welfare of Japan. Informed consent was previously obtained from all patients.

### Preparation of brain sections for FTIRM measurement

Human brain specimens were obtained from the Brain Bank at Tokyo Metropolitan Institute of Gerontology. The medulla oblongata, including LBs from four patients with PD and GCIs from four patients with MSA, were used for measurement. The clinical information of each patient is outlined in Supplementary Table S1.

The samples were unfixed and fresh-frozen according to the procedures for routine tissue processing for pathological and biochemical examination. For each sample from the patient’s brain, 14 µm-thick sections were deposited on CaF_2_ and then immunostained with the anti-human phosphorylated α-syn (Ser129) monoclonal antibody (pSyn#64, Wako) as described previously^10^. The stained sections were then examined by optical microscopy to confirm the presence and locations of LBs and GCIs. Before measurements, these samples were dried at room temperature.

### Synchrotron FTIRM measurement

Synchrotron FTIRM measurements were performed at the infrared beamline BL43IR at the SPring-8 synchrotron radiation facility (Hyogo, Japan) as previously described^10^. A square region (21 µm × 21 µm) including LBs was mapped with an aperture size of 7 µm × 7 µm and 3 µm steps in the horizontal and vertical directions. Because GCIs are generally smaller than LBs, a region (15 µm × 15 µm) including GCIs was mapped with an aperture size of 7 µm × 7 µm and 3 µm steps in the horizontal and vertical directions. Interferograms were acquired with 200 scans, and the signals were averaged. FTIRM was used to generate a spectrum with a nominal resolution of 3 cm^−1^.

### FTIR spectral analysis for 2D mapping

FTIR spectral analysis for amide I was performed using Igor Pro software (version 6.36J, WaveMetrics) as previously described^10^. Total protein distribution was evaluated by calculating the sum of the absorbances at 1540 cm^−1^ and 1640 cm^−1^. The proportion of β-sheet structures was analysed from a curve fit for the FTIR spectra ranging from 1700 cm^−1^–1600 cm^−1^. Spectral data were fitted using four Gaussian species centred at 1628 cm^−1^ and 1680 cm^−1^ (β-sheets) and 1648 cm^−1^ and 1661 cm^−1^ (random coils, α-helices, and others) as previously reported^10^. During the fitting procedure, the peak height was free, whereas the width at half-height was maintained at < 25 cm^−1^.

In the analysis of brain samples, from the spectra acquired in the mapping experiments, the integrated area of the two Gaussian functions representing β-sheets was calculated for each spectrum. After smoothing between adjacent pixels, the result was plotted as a function of the position to produce a contour plot for β-sheets.

### Statistical analysis

Student’s t-test was used for the comparison between LBs and GCIs. The statistical significance was set *P* < 0.001. Statistical calculations were performed with Microsoft Excel 2016 and IBM SPSS statistics ver. 25.

## Supplementary information

Supplementary information.
