# Supplementary material for: The secondary structural difference between Lewy body and glial cytoplasmic inclusion in autopsy brain with synchrotron FTIR micro-spectroscopy
Source: Sci Rep. 2020 Nov 10;10:19423. doi: 10.1038/s41598-020-76565-6 (PMC7656264; doi:10.1038/s41598-020-76565-6)
Supplement: Supplementary file 1 — Supplementary information. [file 41598_2020_76565_MOESM1_ESM.pdf]

Supplementary Information for

The secondary structural difference between Lewy body and glial cytoplasmic inclusion in autopsy brain with synchrotron FTIR micro-spectroscopy

Katsuya Araki [1, 2 *], Naoto Yagi [3], Yuka Ikemoto [3], Hideki Hayakawa [1], Harutoshi Fujimura [4], Taro Moriwaki [3], Yoshitaka Nagai [5], Shigeo Murayama [6] and Hideki Mochizuki [1]

[1] Department of Neurology, Osaka University Graduate School of Medicine, 2-2 Yamadaoka, Suita, Osaka 565-0871, Japan

[2] Toyonaka Municipal Hospital, 4-14-1 Shibaharacho, Toyonaka, Osaka 560-8565, Japan

[3] Japan Synchrotron Radiation Research Institute (JASRI/SPring-8), 1-1-1 Kouto, Sayo, Sayo, Hyogo 679-5198, Japan

[4] Department of Neurology, Toneyama National Hospital, 5-1-1 Toneyama, Toyonaka, Osaka 560-8522, Japan

[5] Department of Neurotherapeutics, Osaka University Graduate School of Medicine, 2-2 Yamadaoka, Suita, Osaka 565-0871, Japan

[6] Department of Neuropathology, The Brain Bank for Aging Research, Tokyo Metropolitan Geriatric Hospital and Institute of Gerontology, 35-2 Sakaecho, Itabashi-ku, Tokyo 173-0015, Japan

Supplementary Table S1

Case	Sex	Age	Cause of death	Clinical diagnosis	Neuropathological diagnosis
**MSA1**	M	76	pneumonia	MSA	MSA
**MSA2**	F	71	aspiration pneumonia	PD?	MSA
**MSA3**	F	85	pneumonia	MSA	MSA
**MSA4**	F	62	unknown	unknown	MSA
**PD1**	M	85	aspiration pneumonia	PD	PD
**PD2**	F	83	shock	PD	PD
**PD3**	F	90	urinary tract infection, sepsis	PD	PD
**PD4**	M	83	pneumonia	PD	PD

Summary of brain tissue samples.

Supplementary Figure S1

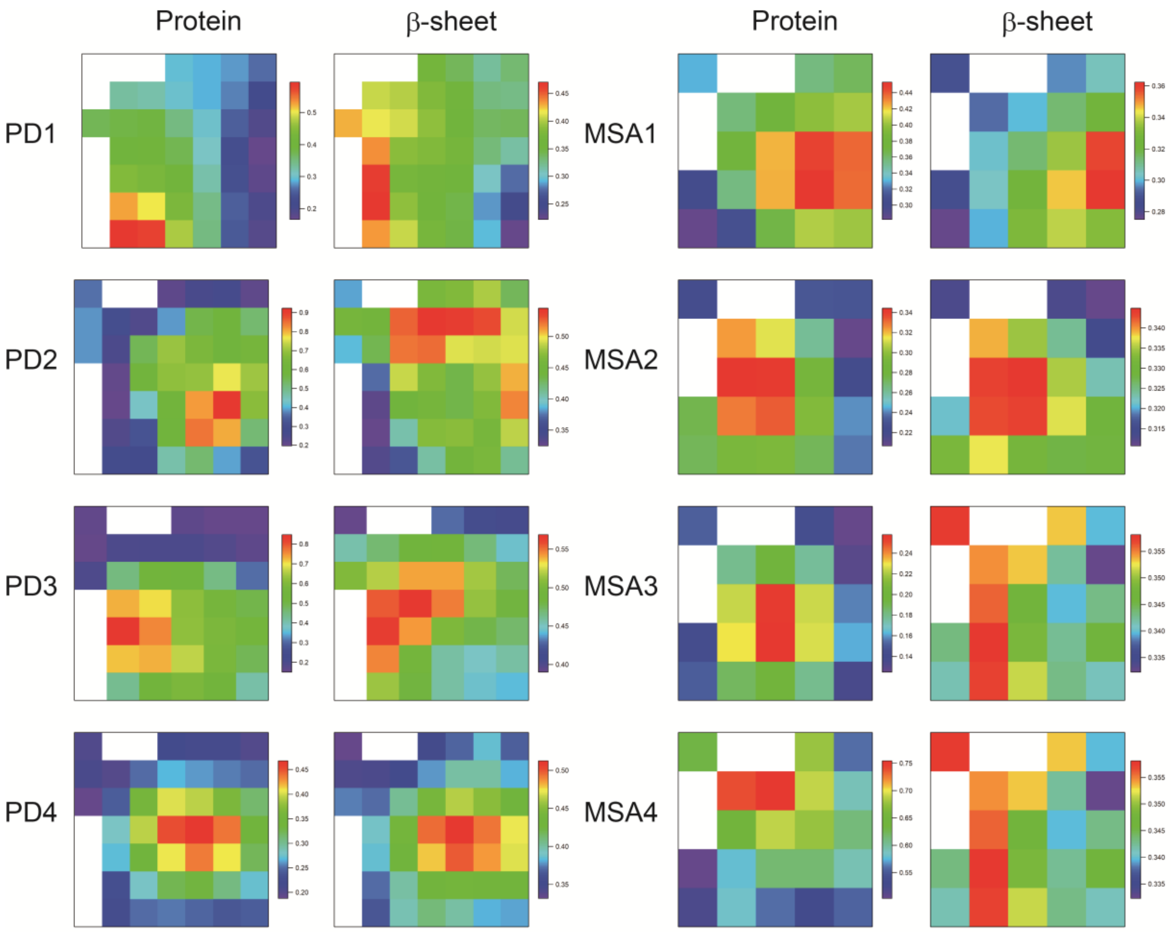

FTIR images of LBs and GCIs in PD and MSA patients. The amount of total proteins, and the proportion of β-sheet structure are mapped. These images are the same as those in Figures 1 and 2 except that they are not smoothed.

Supplementary Figure S2

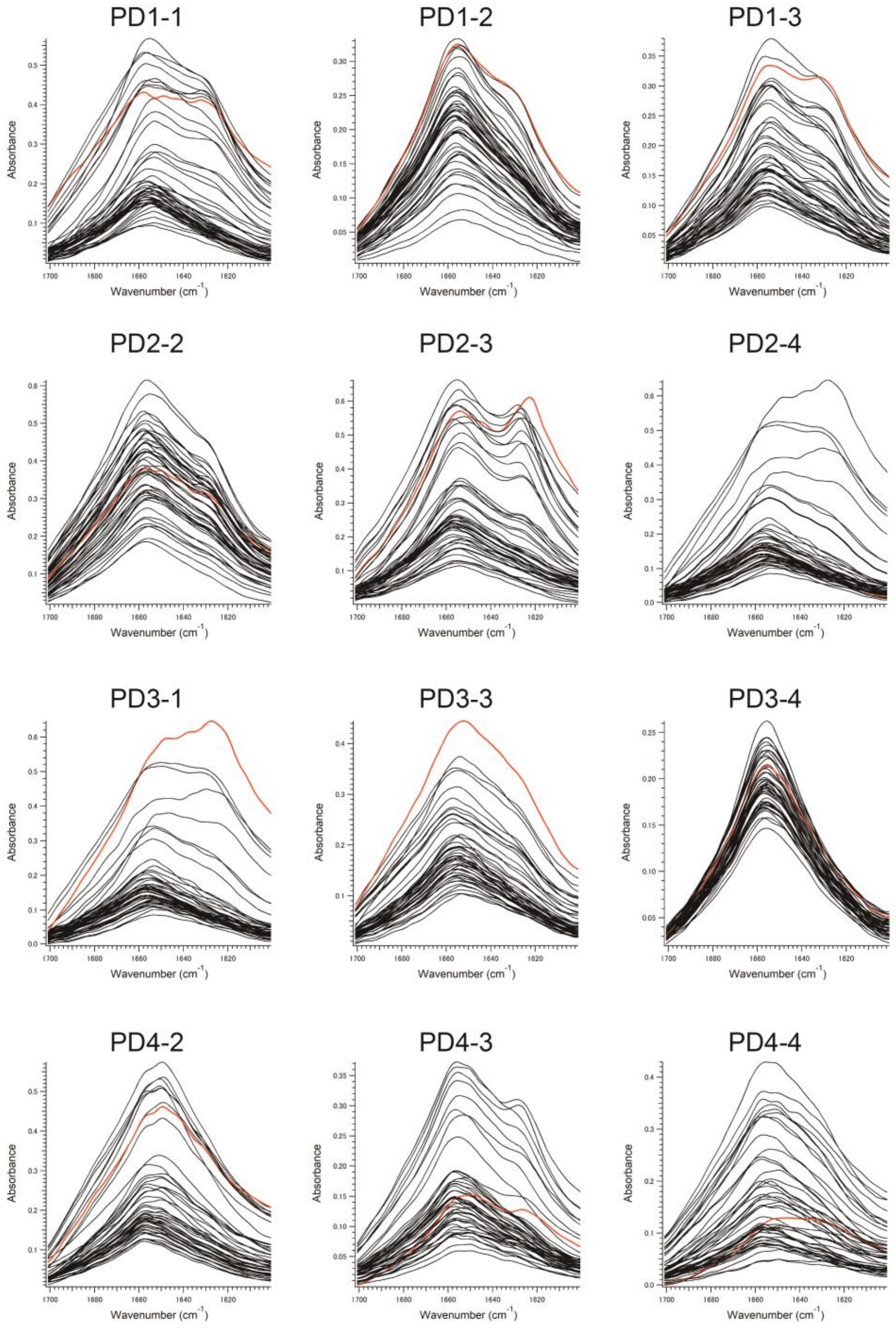

All the FTIR spectra of PD patients. Four LBs were scanned for each patient and a spectrum of one LB is shown in Figure 1. The red solid line in the spectra shows the FTIR spectra of the β-sheet richest point in each LB.

Supplementary Figure S3

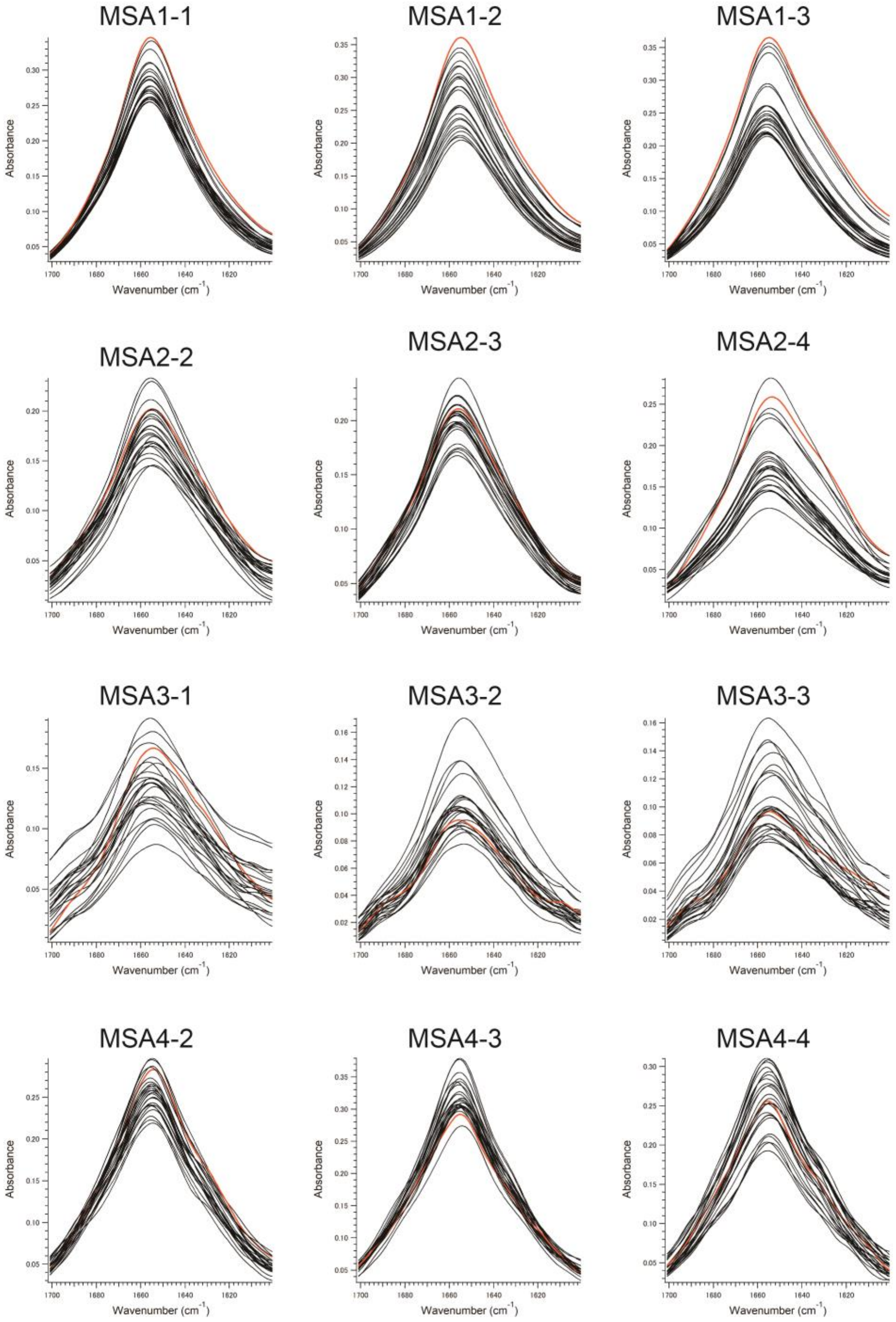

All the FTIR spectra of MSA patients. Four GCIs were scanned for each patient and a spectrum for one GCI is shown in Figure 2. The red solid line in the spectra shows the FTIR spectra of the β−sheet richest point in each GCI.
